# Supplementary material for: Direct measurement of swimming and diving kinematics of giant Atlantic bluefin tuna (Thunnus thynnus)
Source: R Soc Open Sci. 2019 May 8;6(5):190203. doi: 10.1098/rsos.190203 (PMC6549966; doi:10.1098/rsos.190203)
Supplement: Electronic Supplement S2.docx [file rsos190203supp2.docx]

**Electronic Supplement**

**Figure S2**

**Direct Measurement of Swimming and Diving Kinematics of Giant Bluefin Tuna (*Thunnus thynnus*)**

***Adrian C. Gleiss, Robbie S. Schallert, Jonathan J. Dale, Steve G. Wilson, Barbara A. Block***


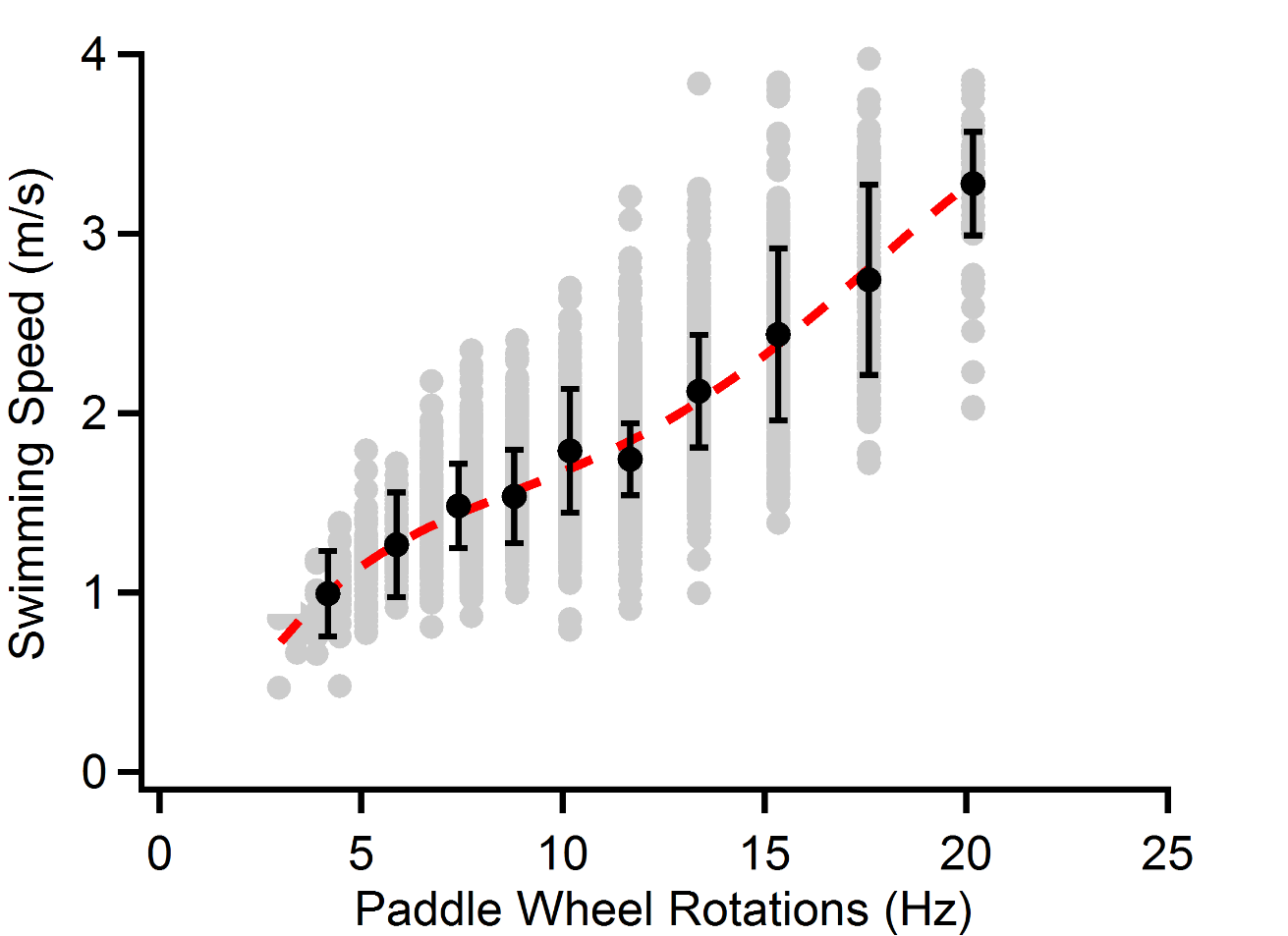


**Fig. S2 Calibration plot for the speed sensor.** Estimated swimming speeds from trigonometry and paddle-wheel rotations determined from the magnetometer (●) were fitted with a fourth order polynomial function (r^2^_=_0.61, ) which was applied to the entire data-set of paddle-wheel rotations to predict speeds. Black symbols (●) represent the binned estimated swimming velocity (±SD) according to rotations of the paddle-wheel rotations.
