# Supplementary material for: Direct measurement of swimming and diving kinematics of giant Atlantic bluefin tuna (Thunnus thynnus)
Source: R Soc Open Sci. 2019 May 8;6(5):190203. doi: 10.1098/rsos.190203 (PMC6549966; doi:10.1098/rsos.190203)
Supplement: Electronic Supplement S1.docx [file rsos190203supp1.docx]

Associate Editor Comments to Author (Dr Denise Greig):

Associate Editor

Comments to the Author:

I would like to commend the authors for the trouble shooting they did to figure out and correct the manufacturer's mistake. I also very much appreciate the clarifications in the method section and the response to reviewers. Like Reviewer #2 notes, there are still a few leftover typos from the first version, but once those are corrected, this will be an interesting addition to the literature on marine animal locomotion.

Below are the typos I noticed:

Line 143. Delete the word “been”

Line 217. Correct “sing” to “using”

**Done**

Line 218. Delete “was”

Line 243. Add “(CWT)” as you use this abbreviation in the next sentence? (if that is the correct interpretation)

**Done**

Line 289-290. Change to “two CATS camera tags” (I think you used the CATS abbreviation above)

**Done**

Line 380. Should this be Fish #1? As everything above was talking about Fish #2? Please be consistent and refer to either Bluefin #1 and #2 or Fish #1 and #2 and use whichever nomenclature you prefer in Figure 3 as well.

**Correct. We have fixed the numbering throughout and now only refer to Fish #x. We have also doubled checked the numbering.**

Table 3. Please add a space and an "s" to "363 descents" in the Table title.

**Done**

**Reviewer comments to Author:**

**Reviewer: 2**

Comments to the Author(s)

I am glad that the authors rectified the speed data. Nonetheless, the paper needs a careful proof-reading, as ‘old’ (wrong) numbers have not been replaced throughout. I have marked a few places, but there can be more. Other than that, I recommend publication. It is a unique set of data.

**We would like to thank the referee again, his thorough review pushed us to turn over all the stones and find the bug after all. We have gone through the MS again and have rectified all old numbers.**

Manuscript

1. L. 327-328: “swimming speed showed a non-linear relationship with tail-beat frequency for Fish #2 (Figure 3).”

**Done**

2. L. 820. Caption to Figure 3. The speed (and stride length) were not updated 3. L. 903. Caption to Figure 6. The speed (2.5 m/s) was not updated

**Done**

3. Supplementary 1. Figure S2 (Supplementary S2) should be replaced. It is the old one.

**Done**

**Data in Dryad**

1. **The abstract seems to reflect the ‘old’ speed calibration: to sustain 3 m/s, a tuna needs more that 1Hz.**
2. **The CATS descent data seems to reflect the old calibration. Please check.**
3. **Some of the columns need units and need explanation. For example, in CATS data: what are Dmin and Dmax? What is the difference between TBF and TBF_Hz? (The latter is the reciprocal of the former, and hence the first TBF is probably the tail-beat period in seconds, and not the frequency). Is TBF in the second file the frequency or the period?**

**4. I would be happier to see representative time series (at least one full period) of yo-yo, slow speed burst-and-coast, high speed burst-and-coast, and steady swimming, rather than the averaged data appearing in Dryad (it can be down-sampled to 10 HZ with hardly any harm). Having both would have been even better.**

Miscellaneous comments

1. L. 282: “Velocity”

**Done**

2. L. 294: “Cats”

**We are not sure what the referee refers to here, if he or she wishes us to write the acronym for the company in not all caps? Since it is an acronym, we have kept it in its current format (CATS) but are of course happy to change it.**

3. L. 778 (Ref. [77]): I am not sure if this reference will pass the production people.

**Indeed. It has been fixed.**

4. Figure 3: I would have added lines of constant stride length v/TBF for a reference.

**That is a good idea. Added. See below.**

5. I would have been more careful in interpretations of yo-yo and burst-and-coast strategies. Both can reduce the cost of transport only if (chemo-mechanical) propulsion efficiency is either thrust- or speed- dependent (Ref. [28], [65] and also the reference cited in footnote 2 of the first review). With constant propulsion efficiency, the swimming strategy that minimizes the cost of transport is constant-speed-constant-depth.

**We appreciate the referees comment here. Indeed, this is still a contentious and exciting field. As the referee correctly points out, unless there is clear difference in the mechano-chemical efficiency between ascent and descent, no energy saving can be realized. Although we admit that experimental data is still scant, but recent experimental data does suggest that drag during active swimming is 2-fold larger than rigid gliding in pacific bluefin (Takagi et al. (2013) J. Theor. Bio). Therefore, mechano-chemical efficiency differs between rigid gliding and undulating swimming. In a general sense, this effect is expected to be small in bluefin as they are very stiff swimmers and may be larger in more carangiform swimmers.**

In the context of these strategies, a few specific comments:

L. 406-410. This is a conjecture. I am not sure if the statement is supported by the data.

**We respectfully disagree. We cite three publications (two of which on the same species studied here), all of which point to the kinematics matching the observed kinematics with energy savings. However, perhaps we should temper the statement about energy savings being the reason for the observed dives. We have altered the statement in the following way:**

*This represents an important aspect of Weihs’ two-stage locomotion model [**17**,* *64**], suggesting that some dives* ***may be*** *performed to increase the economy of horizontal movements, which is in broad agreement with captive work on bluefin tuna [**55**,* *56**].*

L. 435: During acceleration/ascent the fish ‘pays’ for the lack of activity during deceleration/ descent by increasing its TBF, and therefore the frequency argument should probably be reworded. Adding the word ‘red’ before the word ‘muscles’ could solve most of the problem [65].

**Thank you for that suggestion, the type of muscle should have been specified.**

L. 473-476: The difference in energetic costs of ascent and descent is independent of speed. It is just the difference in the potential energy (submerged weight times the change in depth) divided by the propulsion efficiency.

**Agreed in principal, however given that propulsive efficiency is expected to differ substantially between gliding and active swimming ( as revealed but differences in the drag coefficients, see Takagi et al), who categorically states for bluefin: *“The faster the upward swimming speed, the lesser is the kinetic energy saving;”*. We do however, recognise that we need to do our bit to highlight that any savings in mechanical work can ONLY stem from differences in drag between undulating and rigid “swimming”. We have added this to the MS to ensure the reader is aware of the issue:**

XXXXX

L. 493: And also the flex at the caudal peduncle.
